# Mobile phone use and risk of acoustic neuroma

**DOI:** 10.1038/sj.bjc.6603069

**Published:** 2006-03-28

**Authors:** B Hocking

**Affiliations:** 1 Specialist in Occupational Medicine, 9 Tyrone St Camberwell Victoria 3124, Australia

**Sir**,

In order to address the public concern that the use of mobile phones could increase the risk of brain tumours Schoemaker *et al* have conducted a case–control study to assess the risk of acoustic neuroma in relation to mobile phone use in 678 cases and 3553 controls. They concluded ‘there was no association of risk with the duration of use, lifetime cumulative hours of use or number of calls, for phone use overall, or for type of phone’. However, they found an increased risk for tumour ipsilateral to the side most used, for use of phone for 10 years or longer.

When addressing a public concern, the strength of evidence for harmlessness is as relevant as evidence for harm. The study by Schoemaker *et al* (2005) fails to present this.

The quality of the exposure data for the study was entirely dependent on the accuracy of recall by subjects for up to and over 10 years, though this was not substantiated by reference to cross-checking with billing data to determine the margins of error. The accuracy of the recall of this data is crucial but not substantiated in the paper. In fact, the authors refer to the study by Parslow *et al*, which found substantial misclassification in recall data on mobile phone use, but appear not to have not appreciated the significance of this for Type 2 errors and their conclusions.

Parslow *et al* (2003) found only modest correlation between recall data about mobile phone use from 93 volunteers and their outgoing phone bills over a 6-month period, concluding that ‘self reported mobile phone use may not fully represent patterns of mobile phone use’. Similar findings were obtained by Samkange-Zeeb *et al* (2004) and Shum *et al* (2005). These studies found problems in recall data over relatively short periods of time, yet Shoemaker *et al*, place reliance on recall for up to and over 10 years. Inaccurate data will cause substantial misclassification, which will dilute any real effect on acoustic neuroma of cumulative use or number of calls thereby increasing the likelihood of Type 2 error, and considerably diminishes the ability to offer genuine reassurance to the public about the harmlessness of mobile phones. This major limitation is not reflected in the conclusions or abstract of the paper. By contrast the finding of increased acoustic neuroma risk after 10 years of mobile phone use, depending simply on recall of first use, is least likely to be erroneous (Type 1) and is the better substantiated finding in their study.
